# Study Protocol. IDUS – Instrumental delivery & ultrasound. A multi-centre randomised controlled trial of ultrasound assessment of the fetal head position versus standard care as an approach to prevent morbidity at instrumental delivery

**DOI:** 10.1186/1471-2393-12-95

**Published:** 2012-09-13

**Authors:** Deirdre J Murphy, Gerard Burke, Alan A Montgomery, Meenakshi Ramphul

**Affiliations:** 1 Academic Department of Obstetrics & Gynaecology, Trinity College Dublin & Coombe Women & Infant’s University Hospital, Dublin 8, Ireland; 2 Mid-Western Regional Maternity Hospital, Ennis Road, Limerick, Ireland; 3 Primary Care Research, Department of Community Based Medicine, University of Bristol, 25 Belgrave Road, Bristol, BS8 2AA, UK; 4 Coombe Women & Infants University Hospital, Dublin 8, Ireland

**Keywords:** Fetal head position, Second stage of labour, Intrapartum ultrasound, Randomised controlled trial

## Abstract

**Background:**

Instrumental deliveries are commonly performed in the United Kingdom and Ireland, with rates of 12 – 17% in most centres. Knowing the exact position of the fetal head is a pre-requisite for safe instrumental delivery. Traditionally, diagnosis of the fetal head position is made on transvaginal digital examination by delineating the suture lines of the fetal skull and the fontanelles. However, the accuracy of transvaginal digital examination can be unreliable and varies between 20% and 75%. Failure to identify the correct fetal head position increases the likelihood of failed instrumental delivery with the additional morbidity of sequential use of instruments or second stage caesarean section. The use of ultrasound in determining the position of the fetal head has been explored but is not part of routine clinical practice.

**Methods/Design:**

A multi-centre randomised controlled trial is proposed. The study will take place in two large maternity units in Ireland with a combined annual birth rate of 13,500 deliveries. It will involve 450 nulliparous women undergoing instrumental delivery after 37 weeks gestation. The main outcome measure will be incorrect diagnosis of the fetal head position. A study involving 450 women will have 80% power to detect a 10% difference in the incidence of inaccurate diagnosis of the fetal head position with two-sided 5% alpha.

**Discussion:**

It is both important and timely to evaluate the use of ultrasound to diagnose the fetal head position prior to instrumental delivery before routine use can be advocated. The overall aim is to reduce the incidence of incorrect diagnosis of the fetal head position prior to instrumental delivery and improve the safety of instrumental deliveries.

**Trial registration:**

Current Controlled Trials ISRCTN72230496

## Background

Instrumental deliveries are commonly performed in the United Kingdom and Ireland, with rates of 12 – 17% in most centres [1,2]. Knowing the exact fetal head position is a pre-requisite for safe instrumental delivery. Traditionally, diagnosis of the fetal head position is made on transvaginal digital examination by delineating the suture lines of the fetal skull and the fontanelles [3]. However, accurate diagnosis of the fetal head position by transvaginal digital examination can be unreliable [4].

Malpositions in labour in a vertex-presenting fetus are known to be associated with increased prolonged first and second stages of labour, oxytocin augmentation, use of epidural analgesia, chorioamnionitis, assisted vaginal delivery, third and fourth degree perineal lacerations, caesarean delivery, excessive blood loss, and postpartum infection [5-9]. Trial of instrumental delivery in theatre is twice as likely to fail in occipito-posterior (OP) positions and failed trials are associated with increased neonatal and maternal morbidity and trauma [8,10,11].

We propose a randomised controlled trial to evaluate the use of ultrasound to diagnose the fetal head position prior to attempting instrumental delivery.

The hypothesis is that an abdominal ultrasound scan performed in addition to routine clinical assessment reduces the incidence of incorrect diagnosis of the fetal head position which will reduce the risk of maternal and perinatal morbidity.

### Literature review

A search of Medline from 1965 to 2011 and of the Cochrane Library was undertaken, for relevant systematic reviews, meta-analyses, randomised controlled trials, and other clinical studies. The date of the last search was June 2011. We intend to update this before publishing the results of the trial but at the time of finalising the trial protocol, there were no key changes since the search in June 2011. The main keywords used were: instrumental delivery, vacuum, forceps, fetal position, ultrasound, digital examination, randomised controlled trial. In addition, when reviewing published reference lists, key articles cited were also retrieved and reviewed.

The literature relating to the accuracy of digital vaginal examination versus ultrasound as the gold standard is presented in Table 1. Accuracy varied from 20% to 75% [4,12-19]. The authors of a prospective study of a hundred women which set out to evaluate the learning curves of digital examination and transabdominal ultrasound to determine the fetal head position in labour, reported that it was easier to become skilled in ultrasonography than digital examination [19]. Few studies have addressed error rates in ultrasound determined fetal head position among novice ultrasonographers and only two studies have reported error rates of transabdominal scan within a research setting (6.8% and 7.9% respectively) with another study reporting inability to diagnose the fetal head position in 15% [16,17,19]. We found only two studies evaluating the role of ultrasound assessment to determine the fetal head position before instrumental deliveries [20,21]. Akmal *et al.* compared the accuracy of vaginal examination to transabdominal ultrasound examination in 64 women undergoing instrumental delivery and found that vaginal examination was incorrect in 27% cases with errors being more likely with occipito-posterior positions and if the head was at the level of the ischial spines [20]. Wong *et al.* carried out a randomized trial of fifty women undergoing vacuum extraction for prolonged second stage where women were randomly allocated to either digital examination (n = 25) or digital examination together with transabdominal intrapartum ultrasound (n = 25) prior to vacuum extraction by the attending obstetrician [21]. A midwife measured the distance between the centre of the chignon and the flexion point immediately after delivery. The mean distance between the centre of the chignon and the flexion point was 2.1+/−1.3 cm in the group with digital examination and ultrasound assessment and 2.8+/−1.0 cm in the group with digital examination alone, a small but statistically significant difference [21].

**Table 1:** Studies evaluating accuracy of transvaginal digital examination compared to ultrasound in diagnosing the position of the fetal head in labour

**Author, citation**	**Study design**	**Exposures**	**Outcome measures**	**Results**	**Conclusions**
Akmal S et al. J Matern-Fetal Neo M 2002, 12(3): 172-7	Prospective study 496 women in labour (1^st^ & 2^nd^ stages)	DVE vs TAS (gold standard)	Agreement of DVE within ±45° of TAS correct	DVE in agreement with TAS in 163 cases (49.9%)	Digital examination inaccurate in 50% of cases
Souka AP et al. J Matern-Fetal Neo M 2003; 139(1): 59- 63	Prospective study 148 women in labour (1^st^ & 2^nd^ stages)	DVE vs TAS (gold standard)	Agreement of DVE within ±45° of TAS correct	Accuracy of DVE 31.3% in 1^st^ stage & 65.7% in 2^nd^ stage, more likely to be inaccurate in OP position	Digital examination is less accurate than ultrasound, especially in OP position.
Sherer DM et al. Ultrasound Obst Gyn 2002; 19(3): 258-63	Prospective study 102 women in labour (1^st^ stage)	DVE vs TAS (gold standard)		DVE accurate in 24 cases (24%)	High error rate (76%) with digital examination
Sherer DM et al. Ultrasound Obst Gyn 2002; 19(3): 264- 8	Prospective study 112 women in labour (2^nd^ stage)	DVE vs TAS (gold standard)	Absolute error when DVE not consistent with TAS; and inconsistency of >45°	Absolute error of DVE 65% DVE incorrect by > 45° in 44 cases (39%)	Ultrasound improves accuracy
Dupuis O et al. Eur J Obstet Gynecol Reprod Biol 2005; 123(2): 193-7	Prospective study 110 women in labour (2^nd^ stage)	DVE vs TAS (gold standard)	Agreement of DVE within ±45° of TAS correct	In 20% of the cases, DVE differed significantly (>45°) from TAS, higher in OP & OT positions	Transabdominal ultrasound can increase accuracy
Kreiser D et al. J Matern-Fetal Neo M 2001; 10(40): 283-6	Prospective study 44 women in labour (2^nd^ stage)	DVE vs TAS (gold standard)	DVE & TAS findings compared to actual fetal head position at delivery and restitution of the fetal head – if different, considered to be wrong and quantified as =90°, <90° or >90°	TAS less error than DVE: 6.8% vs 29.6%, *p =* 0.011	TAS is more accurate
Zahalka N et al. AJOG 2005; 193(2): 381-6	Prospective study 60 women in labour (2^nd^ stage)	DVE vs TAS vs TVS	Agreement of DVE within 60° of TAS correct	Discrepancy between DVE & TAS 21.7% Discrepancy between DVE & TVS 23.3% 5 cases where DVE erroneously diagnosed position as being OA when it was OP	TAS and TVS more accurate than transvaginal digital examination
Chou R et al. AJOG 2004; 191: 521- 4	Prospective study 88 women in labour (2^nd^ stage)	DVE vs TAS	DVE & TAS findings compared to actual fetal head position at delivery (direct visualisation of position at vaginal delivery after spontaneous restitution of the head or at caesarean section). Considered correct if DVE/TAS within 45° of actual position.	Accuracy of DVE 71.6% vs 92% accuracy for TAS, p = 0.018	TAS more accurate than DVE
Rozenberg P et al. Ultrasound Obst Gyn 2008; 31(3):332 - 7	Prospective study One novice doing both TAS and VE 100 women (≥ 7 cm dilated)	DVE vs TAS	Learning curve of a novice at diagnosis of the fetal head position by DVE & TAS compared to an expert	Error rate of DVE 50% over first 50cases, down to 28% over last cases vs 8% error with TAS	Learning and accuracy of diagnosis of the fetal head position easier & higher with TAS
Akmal S et al. Ultrasound Obst Gyn 2003; 21(5):437-40	Prospective study 64 women undergoing instrumental delivery	DVE vs TAS	Agreement of DVE within ±45° of TAS correct	Error rate of DVE 26.6% (17 cases), igher for OP and OT	DVE inaccurate in a quarter of cases before instrumental delivery
Wong GY et al.	RCT 40 women undergoing vacuum extraction	DVE vs TAS	Accuracy of vacuum cup placement with respect to the flexion point	Mean distance between chignon & flexion point: 2.1 ± 1.3 cm in DVE + TAS group vs 2.8 cm ± 1.0 cm in VE group (p = 0.039)	TAS improves vacuum cup placement

DVE: Digital vaginal examination.

TAS: Transabdominal ultrasound.

TVS: Transvaginal ultrasound.

OA: occipito-anterior, OP: occipito-posterior, OT: occipito-transverse.

### National survey of current practice

We carried out a questionnaire survey in consultant-led maternity units in the United Kingdom and Ireland to establish the current practice of obstetricians with regards to the assessment of women in labour prior to instrumental delivery [22]. Clinical assessment prior to instrumental delivery, factors associated with difficulty in determining the fetal head position, approaches used to enhance determination of the fetal head position, perceived accuracy rates in assessment of the fetal head position and willingness to participate in a clinical trial of ultrasound assessment of the fetal head position prior to instrumental delivery were explored. There were conflicting opinions on the role of abdominal ultrasound in enhancing determination of the fetal head position prior to instrumental delivery, indicating the need for evaluation within a randomised controlled trial [22]. More than half the obstetricians agreed that there was a need for a trial and would participate in such a trial.

### Validation study

Prior to starting this study, it was important to compare the accuracy of diagnosis of the fetal head position in the second stage of labour by ultrasound scan performed by a novice sonographer and by clinical assessment, to that of an expert sonographer (gold standard); and to evaluate the acceptability of ultrasound in the second stage of labour to women and clinicians [23]. We recruited sixty women who had: (i) an abdominal scan performed by a novice; (ii) an abdominal scan performed by an expert ultrasonographer; and (iii) a clinical assessment performed by an obstetrician or midwife; in the passive second stage of labour. Each assessor was blinded to the findings of the others. The ultrasound findings of the novice and expert ultrasonographer were consistent in 52 (87%) cases for the fetal head position and the novice made no occipito-anterior/occipito-posterior (OA-OP) errors. The clinical diagnosis of the fetal head position was incorrect in 25 (42%) cases with 8 (13%) OA-OP errors [23]. We used these findings as an estimate of the primary outcome for the power calculation, Women and clinicians did not consider the ultrasound assessment to be intrusive. In summary, we found that an abdominal scan by a novice ultrasonographer is an accurate and acceptable method of diagnosing the fetal head position in the second stage of labour [23].

#### Aims and objectives

The aim of this study is to compare routine clinical assessment of the fetal head position alone versus clinical and ultrasound assessment of the fetal head position prior to instrumental delivery.

### The primary outcome is to compare the incidence of incorrect diagnosis of the fetal head position

The secondary outcomes are:

to compare the incidence of neonatal trauma, low Apgar scores, fetal acidosis or admission to the neonatal unit

to compare the incidence of primary postpartum haemorrhage, third and fourth degree perineal tears or prolonged postnatal admission

to compare the incidence of sequential use of instruments, instrumental delivery with more than one operator, failed instrumental delivery, transfer to theatre or caesarean section

to compare the decision-delivery intervals

## Methods

### Recruitment and intervention

Recruitment of women to the study will follow a three stage process.

(1) All potentially eligible women will be given written information about the study in the antenatal clinic. A leaflet and covering letter will explain the trial purpose and design, making it clear that women will only become eligible for the study if they require an instrumental delivery. The leaflet will contain contact details to allow women to discuss the study further if they wish.

(2) Once a woman has presented in early labour or for induction of labour, a research fellow will seek written informed consent if the following criteria are satisfied:

i) the midwife looking after the woman assesses her to be capable of providing informed consent.

ii) the woman has adequate pain control.

iii) the woman has not used systemic opiates in the last four hours.

(3) Once consent has been given the mother will not be consulted again unless she requires an instrumental delivery. After confirmation that all criteria are met, the research midwife/fellow will obtain the allocation.

#### Inclusion criteria

This study will be limited to nulliparous women at term (≥37 weeks' gestation) with singleton cephalic pregnancies, aiming to deliver vaginally who require an instrumental delivery in the second stage of labour.

#### Exclusion criteria

Women with a contraindication to instrumental delivery, or who have a limited understanding of English or are under 18 years of age. Eligibility will also be at the discretion of the responsible obstetrician in cases where there is urgency due to suspected fetal compromise (“fetal distress”).

### Allocation to trial groups

Allocation of eligible women who consent to participate in the trial will be concealed using a fully automated centralised web-based system provided by the Bristol Randomised Trials Collaboration. The randomisation sequence will be created by using block sizes of 4, 8 and 12 and stratified by centre, in a 1:1 ratio for usual care versus intervention.

### Intervention

#### Usual care arm

**Figure 1:**
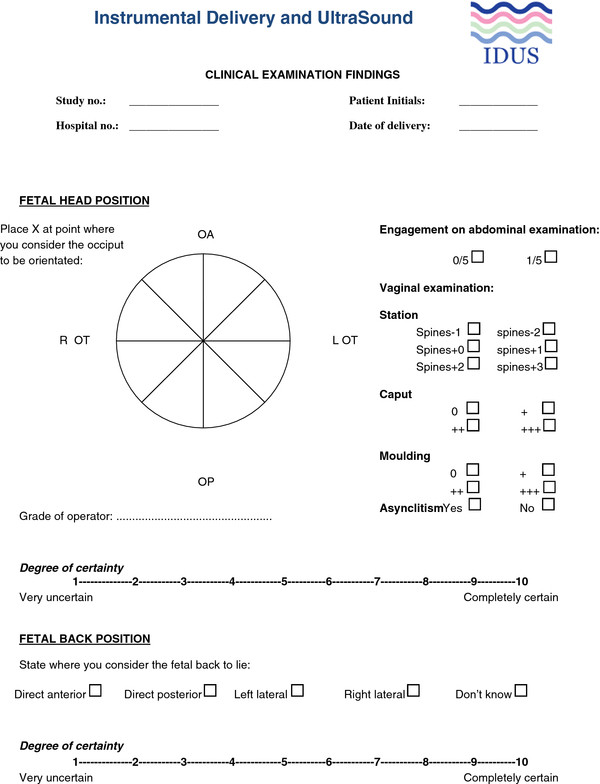
Data sheet: digital vaginal examination findings.

Women allocated to receive usual care will be managed according to Royal College of Obstetricians and Gynaecologists (RCOG) guidelines and the local hospital protocol [3]. The women will be assessed by abdominal and digital vaginal examination prior to instrumental delivery. Following clinical assessment, location of the fetal occiput in relation to pelvic landmarks will be indicated visually by way of a cross on a data sheet depicting a circle, like a clock, divided into 24 sections, each of 15 degrees (Figure 1). The position will then be classified as OA for direct occipito-anterior, ROA and LOA for right and left occipito-anterior respectively; OP for direct occipito-posterior, ROP and LOP for right and left occipito-posterior; ROT and LOT for right and left occipito- transverse respectively. The obstetrician may then proceed to instrumental delivery. The full clinical assessment, delivery procedure, delivery outcome and measures of early morbidity will be recorded on a standard instrumental delivery proforma. The mother and the neonate will be followed-up until hospital discharge.

#### Intervention arm

Women in the intervention group will be managed in the same way. In addition they will receive an ultrasound scan to assess the position of the fetal head and spine. Immediately before or after the clinical examination and before application of the instrument, the fetal head position will be determined sonographically by a trained research fellow. The research fellow will be trained before the start of the trial by a consultant sub-specialist in fetal and maternal medicine and perform a minimum of thirty five ultrasound assessments in the second stage of labour as described in the validation study [23]. For all ultrasound assessments, image- directed pulsed Doppler equipment (Sonosite Titan) with a multifrequency sector array transabdominal transducer, and a 3.5 MHz sector ultrasound probe, will be used.

With the patient in a supine position the ultrasound transducer will first be placed transversely over the maternal abdomen and moved longitudinally to identify landmarks for the fetal spine and head position as described in the validation study [23]. The transabdominal probe is then rotated through 90 degrees, to obtain the transverse view of the fetal spine. Following this, a sliding motion towards the fetal head will be made to obtain a view of the following midline fetal cranial structures: midline cerebral echo, falx cerebri and thalamus and anterior or posterior cranial structures including the orbits and nuchal region. The fetal head position will then be classified as previously described. The obstetrician will be informed of the ultrasound findings to facilitate decision making and may then proceed to instrumental delivery.

### Outcome measures

#### Primary outcome

The primary outcome measure is incorrect diagnosis of the fetal head position. Most errors of clinical diagnosis are where the position is classified as occipito-anterior but is in fact occipito-transverse or occipito-posterior. Incorrect diagnosis of the fetal head position will be established according to any of the following criteria:

i) Position of the head at the time of delivery

If the position of the fetal head was classified as occipito-anterior and is delivered occipito-posterior the diagnosis of the fetal position will be considered incorrect.

ii) Instrument markings on the neonatal head and face

**Figure 2:**
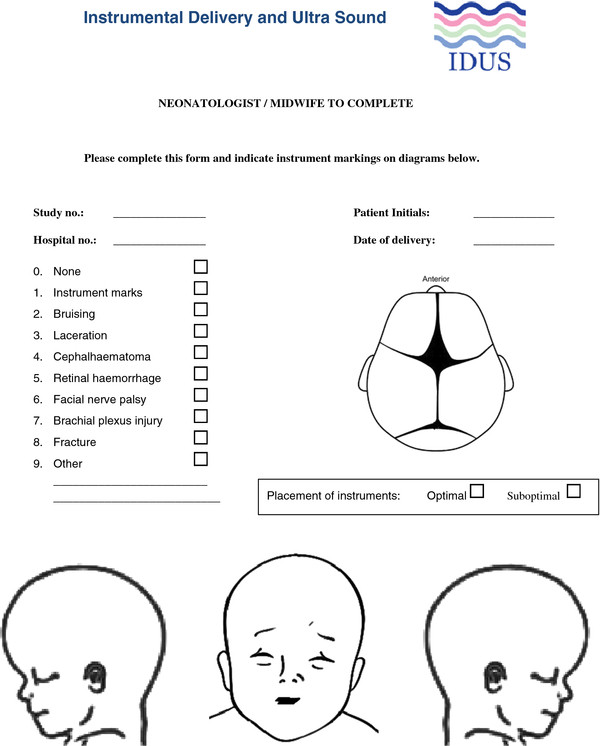
Data sheet: instrument markings on neonatal head and face.

The neonatologist or midwife who attends the delivery will examine the baby. He/she will be asked to record the markings of the instrument on a drawing of the head and lateral aspects of the face (Figure 2). The recorded markings will be used to indicate misplacement of the instrument at a distance from the flexion point (vacuum) or over the face (forceps). If for example the recorded position prior to instrumental delivery was occipito-anterior and the instrument placement suggests an occipito-transverse or occipito-posterior position the diagnosis of the fetal position will be considered incorrect. Furthermore, the diagnosis will be considered incorrect if the markings are more than 45^0^ from the documented fetal head position.

iii) Position at caesarean section

If the delivery is completed by caesarean section the operator will record the position of the head at delivery. If the position of the fetal head was defined as occipito-anterior but is in an occipito-posterior position at caesarean section the diagnosis of the fetal position will be considered incorrect. This information will be cross-referenced with the instrument markings recorded by the neonatologist in cases where there was an initial attempt at instrumental delivery.

The primary outcome will be validated independently by a single investigator who is not involved in scanning or the delivery by reviewing the trial documents (fetal head position recorded by the obstetrician, diagrammatic records of instruments markings on the neonate and documented position of the head position at delivery as described above). Trial allocation will be concealed from this person. Two additional data items will be recorded; i) where the position has been correctly identified the application of the instrument will be classified as optimal or sub-optimal based on the instrument markings and ii) where there is discordance between the findings of the clinician and ultrasonographer the researcher will record whether the ultrasound finding was accepted or not.

#### Secondary outcomes

Secondary neonatal outcomes will include trauma, low Apgar scores, low arterial blood gases and admission to the neonatal intensive care unit (NICU). Neonatal trauma will include bruising, laceration, cephalhaematoma, retinal haemorrhage, facial nerve palsy, brachial plexus injury and fractures. Paired cord blood gases will be taken routinely to measure arterial and venous pH and base excess. Arterial pH below 7.10 and base excess greater than −12.0 mmol/l will be used as the threshold to define significant fetal acidosis.

Secondary maternal outcomes will include extensive perineal tearing involving the anal sphincter (third or fourth degree tears), postpartum haemorrhage, shoulder dystocia, and length of postnatal hospital stay. Primary post partum haemorrhage is defined as an estimated blood loss at delivery and in the first 24 hours of more than 500mls. Postnatal stay will be considered prolonged if more than 3 days’ duration. Maternal and neonatal complications will be defined clinically according to the attending clinicians.

Procedural issues will be recorded in terms of place of delivery, need for senior obstetric support, transfer to theatre, use of sequential instruments, failure of instrumental delivery or proceeding directly to caesarean section and the decision to delivery interval.

#### Follow-up

Clinical follow-up of the mother and neonate will be completed prior to hospital discharge.

#### Trial end

The trial will be considered complete after the final review of the last subject participating in the trial. Trial completion will be notified to the Competent Authority and the Ethics Committee using the appropriate form.

#### Statistical analysis

Data analysis and reporting will proceed according to CONSORT guidelines for randomised controlled trials, and will be conducted blinded to group status by the trial statistician and researcher. The first stage of analysis will be to use descriptive statistics to describe recruited individuals in relation to those eligible, and to investigate comparability of the trial arms at baseline. The primary analysis will involve an intention-to-treat comparison between the two groups for the primary outcome adjusted for stratification/minimisation factors – this will include study centre. Secondary outcomes will be analysed in a similar way. All analyses will use appropriate (that is, logistic or linear) regression models, with results presented as point estimates (odds ratios or difference in means), 95% confidence intervals and p values. Further secondary analyses will involve planned subgroup analyses and will use multivariable regression models with appropriate interaction terms to ascertain any differential effects in relation to, choice of instrument and operator experience.

#### Feasibility

We have completed a prospective cohort study and multi-centre randomised controlled trial comparing restrictive versus routine use of episiotomy at instrumental delivery [24,25]. Interventional studies in the second stage of labour require great sensitivity in terms of appropriate recruitment, randomisation and follow-up. A large number of women need to be approached in the antenatal period of whom only a small proportion will ultimately be invited to participate in the trial. The number of women who are eligible but not recruited needs to be recorded. Obstetricians and midwives are under pressure when planning instrumental delivery in the second stage of labour which needs to be taken in to consideration when designing a second stage clinical trial. Our research team has extensive experience of performing studies in this context and the proposed study design and sample size reflects an accurate estimate of what is feasible within the proposed time frame and available resources.

#### Sample size

The rate of inaccurate diagnosis (difference of more than 45 degrees) was hypothesized to be 20% in the usual care arm (clinical assessment alone) and 10% in the intervention arm (clinical assessment and ultrasound). With 225 women per arm, the study will have 80% power with 5% two-sided alpha, to detect the hypothesized 10% difference. However, it is possible that inaccuracy rates are being under- or over- estimated. It could be argued that any difference in effect on the primary clinical outcome would be worth detecting. Rather, given the need for timely delivery of evidence, we have specified detectable differences for realistic sample sizes recruited within a reasonable time frame within the constraints of the available funding.

The combined annual birth rate for the two recruiting hospitals is 13,500, and around 40% of women will be nulliparous, of whom 30% will have an instrumental delivery. We estimate that there will be a total of 3240 instrumental deliveries among nulliparous women over the 24 month recruitment period based on hospital statistics for 2007. Allowing for 30-50% exclusion and non-consent, 95% collection of the primary outcome, and recruiting for 24 months during office hours yields a conservative estimate of 450 participants (225 per arm) for analysis. This would enable detection of a between-group difference of 10–13 percentage points (odds ratio 0.44 to 0.55) with 80% power and 5% two-sided alpha, and would certainly be considered by women and clinicians as worthwhile. These conservative recruitment estimates take account of eligibility criteria, non-english speaking women and the potential difficulty of randomisation and ultrasound evaluation in the context of suspected “fetal distress”.

#### Timetable

Total 36 months: Commencing 01.12.10 - Estimated completion date 31.12.13

1–6 months: regulatory approvals, pre-trial ultrasound training and pilot study

7–31 months, recruitment, intervention, data collection

32–36 months, data analysis/reporting; Peer-review publications; Presentations.

### Governance issues

#### Ethical committee permission

Ethical committee approval from the Coombe Women & Infants University Hospital and the Mid-Western Regional Maternity Hospital, Limerick, have been granted for this study.

#### Data management

Data will be collected on a case report form (CRF) at the time of recruitment by a trained researcher. The researcher will also be responsible for ensuring that the details of the delivery are recorded and documented according to the study protocol. The inpatient maternal and neonatal notes will be marked so that they can easily be recovered following discharge from hospital. After discharge the CRF will be collected by the local co-ordinator and the completeness of the data checked against the woman’s and neonate’s notes. Any errors will be followed up at this time. The data will be entered into a computer database (password protected) at the Coombe Women and Infants University Hospital.

#### Trial management group (TMG)

This group will be in charge of the everyday running of the trial. The full group will meet 4-monthly and as required. Day-to-day decision making will be by Prof. Deirdre Murphy, Dr. Gerard Burke and the trial researcher, with meeting of the full committee as above.

#### Trial steering committee

A trial steering committee will be set up which will have overall supervision of the trial. It will meet prior to commencement of the trial and then at least 6 monthly until completion. A meeting of the TSC will be held within a month of every DMEC meeting to consider their recommendations. An independent Chair will be sought for the TSC.

#### Data monitoring and ethics committee (DMEC)

An independent safety and data monitoring committee will also be formed. They will meet 6-monthly. They will advise the Trial Steering Committee (TSC) on the need for continuing or stopping the trial.

#### Safety considerations

Serious adverse events (SAEs) will be recorded and reported to the regulatory authorities. SAEs include maternal death, surgery (other than caesarean section) admission to intensive care unit or perinatal death. In the event of a SAE occurring, a form will be completed by the local researcher and faxed to the trial co-ordinating centre at the Coombe Women’s Hospital within 72 hours. The chair of the Data Monitoring and Ethics Committee (DMEC) will be informed and the Chair of the MREC will also be informed by the DMEC Chair if considered appropriate.

## Discussion

### Potential and implementation of the findings

It is both important and timely to evaluate the use of ultrasound to diagnose the fetal head position prior to instrumental delivery. To date most studies relating to the use ultrasound for diagnosis the fetal head position have been observational studies with only one small randomised controlled trial found and although the results are promising, this use of ultrasound needs to be formally evaluated within the setting of a clinical trial before its routine use can be advocated.

Misdiagnosis of the fetal head position at digital vaginal examination is more likely with the clinically important malpositions, especially OP, leading to attempts to deliver vaginally with incorrectly applied instruments. Furthermore, uncertainty about the fetal head position may be a factor in the decision to transfer more women to theatre for a trial of instrument, causing in some cases an unnecessary delay in delivery and wasting valuable theatre time; equally failed instrumental delivery in a labour room due to incorrect diagnosis of the fetal head position is associated with increased physical and psychological morbidity. Accurately diagnosing a malposition of the fetal head prior to instrumental delivery may lead to appropriate use of senior support, transfer to theatre for trial of instruments or indeed abandoning the procedure in favour of a caesarean section. The overall aim is to reduce the incidence of incorrect diagnosis of the fetal head position prior to instrumental delivery and improve the safety of instrumental deliveries.

### Dissemination

We aim to raise awareness of this clinical question and our proposed research approach at local, national and international meetings. A final report will be prepared for the funding body and papers will be prepared for peer-review publication and national/international dissemination.

## Competing interests

All authors and their relations have no financial connections with companies that may have an interest in the submitted work, and no non-financial interests that may be relevant to the article.

## Authors’ contribution

Prof D.J. Murphy and Dr G. Burke had the original idea for the trial. Prof D.J. Murphy, Dr A. Montgomery and Dr M. Ramphul designed the trial. Prof D.J. Murphy and Dr M. Ramphul drafted the paper which was revised by all authors. Prof DJ Murphy is the guarantor. All authors read and approved the final manuscript.

## Details of ethics approval

We received ethical approval from the Ethics Research Committee in the Coombe Women & Infants University Hospital on the 5^th^ October 2010 and from the Ethics Research Committee in the Mid-Western Regional Maternity Hospital, Limerick on the 9^th^ August 2011. Women will provide written consent.

## Funding

The trial is being funded by the Health Research Board of Ireland (grant reference number POR/2010/55). The funding source had no role in the trial design, writing of the report, or the decision to submit the paper for publication.

## Pre-publication history

The pre-publication history for this paper can be accessed here:

http://www.biomedcentral.com/1471-2393/12/95/prepub
